# Diseases of Respiratory Organs

**Published:** 1905-10-28

**Authors:** 


### DISEASES OF RESPIRATORY ORGANS.

**Influenza.**—The influenza bacillus has attacked the civilised world during the last two centuries about once every fifteen years, and each time seems to have died out rapidly until the last invasion, when it became a permanent inhabitant. Considering the various evils it causes, it is remarkable how little work has been done on its life history and the possibility of destroying it. The Chicago [1] health authorities, noticing a rise in the death-rate of 45 per cent. from heart disease and from pneumonia since the arrival of influenza, have planned a campaign against it, urging the isolation of patients, the disinfection of clothing, and especially the bacterial investigation of suspicious cases. They have been able to correct the diagnosis in various cases and to show the presence of the bacillus as a complication of several diseases where it was unsuspected. It has indeed been argued that the increase in the pneumonia death-rate is due to the inclusion of very young or old cases and that influenza has therefore had no effect. W. H. Park [2] remarks that, like pneumococci and the streptococci, influenza bacilli are frequently found in the throat, especially at some seasons, and that it is usually difficult to decide to which organism the symptoms are due. Hence he thinks microscopic investigations have only a theoretical interest. Unless the resistance is lowered, or a specially virulent strain of organism is present, no symptoms of disease are set up. The influenza bacillus seems, indeed, to have the power of starting an acute form of conjunctivitis which, in the case of infants, may lead to perforation of the cornea. E. B. Deuch also speaks of the frequency in influenza of aural complications, in which the necessity arises of incising the tympanum, and if that fails, of opening the mastoid process.

**Pneumonia.**—J. Lindsay Steven [3] discusses the question whether lobar pneumonia is a general disease " the brunt of which may fall on any part " of the body, or a specific fever with a local lesion, the site of which is in the lung. Washbourn and Kingston Fowler have published cases of pneumococcal pleurisy with constitutional symptoms like those of pneumonia, and Fowler states that he has seen many cases diagnosed as pneumonia, in which post-mortem the only lesion was an acute pleuritis. Although Steven has some difficulty in accepting their views, he gives an account of a case of acute pulmonary disease with a history similar to that of lobar pneumonia, but with the physical signs of pleurisy, with effusion, and fatal on the sixth day. A pint of opaque serous fluid containing the pneumococcus was drawn off during life and the temperature ran up to 107°. In the absence of a post-mortem there may have been a true pneumonia also, but the pleurisy which accompanies the early stages of pneumonia does not usually cause a fluid, but a fibrinous exudate, though purulent effusions in later stages are not uncommon. The case then is one of acute pneumococcal pleurisy, fatal before the exudate became purulent, but otherwise not unlike the instances of pneumococcal peritonitis and arthritis which have been recorded by various writers. It is certain that in some of these no pulmonary affection takes place, and probably some cases of pneumococcal pleurisy do not affect the lung tissues at all.

[1] Lancet, April 15. [2] Med. Rec., March 18. [3] Glasgow Med. Jour., May.

*(To be continued.)*

